# Study of Lysozyme Activity in Bird Egg Whites by Fluorescence Polarization Assay Using Chitooligosaccharide Tracer

**DOI:** 10.3390/foods14081365

**Published:** 2025-04-15

**Authors:** Liliya I. Mukhametova, Dmitry O. Zherdev, Anton N. Kuznetsov, Olga N. Yudina, Sergei A. Eremin, Vadim B. Krylov, Nikolay E. Nifantiev

**Affiliations:** 1 Faculty of Chemistry, M.V. Lomonosov Moscow State University, Leninsky Gory 1/3, 119991 Moscow, Russia; liliya106@mail.ru (L.I.M.); me@zherderini.ru (D.O.Z.); eremin_sergei@hotmail.com (S.A.E.); 2 Laboratory of Glycoconjugate Chemistry, N.D. Zelinsky Institute of Organic Chemistry, Russian Academy of Sciences, Leninsky Prospect 47, 119991 Moscow, Russia; olgay05@mail.ru; 3 Laboratory of Synthetic Glycovaccines, N.D. Zelinsky Institute of Organic Chemistry, Russian Academy of Sciences, Leninsky Prospect 47, 119991 Moscow, Russia; antonqzn@gmail.com

**Keywords:** lysozyme, chitinase activity, egg white, fluorescence polarization, chitooligosaccharide

## Abstract

The storage duration and hatchability of eggs largely depend on the lysozyme content in egg whites; therefore, determining the lysozyme status is important for characterizing their quality. For the first time, a fast and accurate method for determining the active lysozyme in egg whites has been proposed to establish the lysozyme status of eggs using the fluorescence polarization assay and synthetic chitooligosaccharide conjugates with a fluorescent label without sample preparation. The egg whites of hens, black hens, chukars, quails, ducks, geese, turkeys, peacocks, and ostriches were studied. Samples of egg whites from hens, black hens, chukars, and quails demonstrate the possibility of measuring the lysozyme activity. Samples of hen and black hen eggs from a farm showed approximately the same enzymatic activity of lysozyme. A relatively higher enzymatic activity was demonstrated by the samples from quail egg whites; however, a wide range of data was observed among the eggs. Chitooligosaccharide conjugates demonstrate that they bind only to C-type lysozyme, and no interaction with G-type lysozyme has been shown. Lysozyme activity in the egg whites of duck, goose, turkey, peacock, and ostrich eggs has not been detected by using the obtained chitooligosaccharide tracers, which may be related to the structural features of lysozyme in different bird species. Thus, the method of fluorescence polarization (FP), using fluorescently labeled chitopentaoside to determine the lysozyme status, can be used to characterize hen, black hen, chukar, and quail eggs, which will allow for the selection of a batch of eggs with a high content of active lysozyme, for example, for long-term storage.

## 1. Introduction

Currently, significant attention is being paid to simple and rapid methods of analysis that do not require bulky, expensive equipment and can be performed virtually anywhere using portable devices. One such method is the fluorescence polarization assay (FPA). The principle of the FPA is based on the change in the rotational diffusion rate for a fluorescently labeled molecule when it binds to a molecule of a higher molecular weight [1,2]. The FPA has been applied for the analysis of both low [1,3] and high molecular weight compounds [4,5], the diagnosis of infectious diseases [6], the investigation of biomolecular interactions in solutions [7,8,9], and the quantification of enzyme activity [2,10].

Lysozyme, a crucial enzyme also known as muramidase (EC 3.2.1.17), is a hydrolase primarily found in egg whites, tears, and various bodily secretions of eukaryotes cells [11]. Its classification extends beyond the simple “hydrolase” designation; it is a glycoside hydrolase, specifically targeting β-(1→4) glycosidic bonds [12,13,14,15]. This specificity allows it to break down peptidoglycans, the primary component of bacterial cell walls, and chitin, a structural polysaccharide in fungal cell walls and exoskeletons of arthropods. The diverse array of lysozyme types—including C-type, G-type, I-type, plant-type, bacterial, and bacteriophage-derived lysozymes—reflects the enzyme’s broad evolutionary distribution and functional adaptation across different organisms [16,17]. These variations often result in subtle differences in substrate specificity and catalytic efficiency. For example, hen egg white lysozyme (HEWL), a well-studied C-type lysozyme, displays a higher affinity for certain bacterial peptidoglycans than others, influencing its antimicrobial effectiveness. The mechanism of the lysozyme action involves binding to the substrate and catalyzing hydrolysis via a two-step process, often utilizing a combination of acid–base catalysis and substrate distortion. The enzyme’s active site perfectly complements the shape of the substrate, effectively weakening the glycosidic bond before cleaving it. This enzymatic breakdown compromises bacterial and fungal cell wall integrity, leading to cell lysis and death. Consequently, lysozyme plays a pivotal role in innate immunity, providing a first line of defense against pathogenic infections in mammals and other organisms [15,18,19,20]. Its antimicrobial properties have also led to its exploration as a therapeutic agent, particularly in wound healing and topical applications against bacterial infections. Further research continues to explore its potential applications in food preservation and various industrial processes, leveraging its efficient and environmentally friendly antimicrobial capabilities.

Its concentration is important for the clinical parameters for disease progression [21]. In addition to its enzymatic activity, the capacity of lysozyme to interact with various antigenic molecules is currently being actively investigated [22]. Thus, the lectin-like ability of lysozyme to recognize bacterial carbohydrate antigens without lytic activity was reported for the tetrasaccharide related to the lipopolysaccharide of *Klebsiella pneumoniae* [23]. The FPA was developed to study the activity of type-C lysozyme for diagnostic purposes, using fluorescently labeled natural peptidoglycan [24,25]. However, the use of a natural substrate makes it difficult to compare the activities of enzyme samples obtained at different times. To standardize the method for studying lysozyme activity, fluorescently labeled glycoconjugates were synthesized and a method for determining the enzymatic activity of lysozyme was developed [26]. This method is based on the rapid formation of a complex of the enzyme with a synthetic chitooligosaccharide tracer (resulting in an increase in the FP signal) followed by the lysozyme-induced destruction of the complex (leading to a decrease in the FP signal over time). This technique was validated by measuring the activity of lysozyme in human tears [26] and in chicken egg protein [27]. It is of interest to explore the possibility of measuring the lysozyme activity of egg proteins of different bird species. The lysozyme content in egg whites is of great importance both for their storage and for hatchability. The establishment of the lysozyme status of eggs will allow them to be selected for long-term storage or for breeding chicks. In this study, we applied the FP method and examined lysozyme activity in egg proteins from chukars (*Alectoris chukar*), quails, ducks, geese, turkeys, peacocks, and ostriches and compared the results with the activity of lysozyme in hen eggs. Since the proteins of these eggs contain lysozyme of two types, C (chickens, chukars, quails, ducks, turkeys, and peacocks) and G (geese and ostriches), the possibility of using fluorescent synthetic chitooligosaccharide conjugates to determine the activity of lysozyme in the egg white of different breeds of birds will be shown.

## 2. Materials and Methods

### 2.1. Reagents and Materials

Tracer **1** and **2** were obtained by conjugation of spacered chitotrioside and chitopentaoside [26,27] with fluorescein.

Eggs of white (*n* = 10) and black hens (*n* = 10), chukars (*n* = 10), quails (*n* = 20), ducks (*n* = 10), geese (*n* = 10), turkeys (*n* = 10), peacocks (*n* = 1), and ostriches (*n* = 1) were purchased at a local farm. The following commercially available reagents were used: hen egg lysozyme (Sigma, Darmstadt, Germany), Na_2_HPO_4_, NaH_2_PO_4_, and NaCl (Sigma-Aldrich, Darmstadt, Germany).

### 2.2. Polarization Fluorescence Analysis

A Sentry-200 portable instrument (Ellie LLC, Germantown, WI, USA) was used to perform FP measurements in a borosilicate glass tube (10 × 75 mm) with λ_ex_ = 485 and λ_em_ = 535 nm. The Sigma Plot 11 software program (Systat Software Inc., Palo Alto, CA, USA) was used to process the acquired data.

Fluorescein-labeled 5-oligosaccharide tracer working solutions (TWSs) were made in 25 mM phosphate buffer with 0.15 M of NaCl (pH 7.4). The solutions’ fluorescence intensity, which was roughly 220,000 units and matched a tracer concentration of 3 nM, was ten times greater than the background signal. The FP signal change was measured every 20 s for 10 min after 100 μL of egg white samples were added to 900 μL of TWS after they had been diluted ten times in 25 mM phosphate buffer, which contained 0.15 M of NaCl with a pH of 7.4. Each experiment was repeated at least three times. The following equation was used to approximate the obtained FP dependences on time for various lysozyme samples and to calculate k (the observed rate of dissociation of tracer with lysozyme) as a parameter reflecting lysozyme activity.

mP = mP_0_ + (mP_max_ − mP_0_) e ^−kx^ (1)

## 3. Results

**Figure 1:**
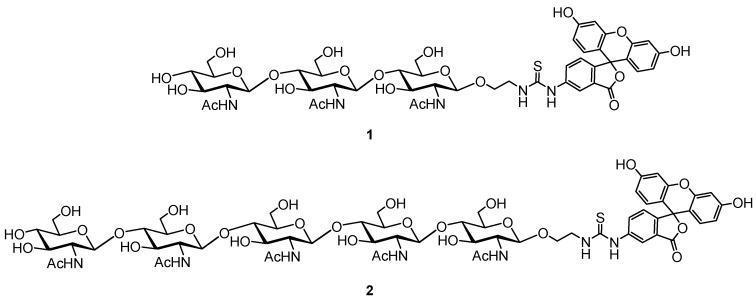
Structure of FITC-labeled tri- (**1**) and pentasaccharide (**2**) tracers.

The proposed rapid FP method, utilizing fluorescently labeled oligosaccharides **1** and **2** (Figure 1) for the lysozyme activity assessment, was successfully validated for measuring the activity of human and chicken C-type lysozyme [26,27]. Synthetic tracers **1** and **2** were used to assess the lysozyme activity in the hen egg white. This study was conducted to evaluate the possibility of measuring the lysozyme activity in egg whites from other bird species. Lysozymes from different avian species are known to possess highly conserved amino acid sequences; that is why their enzymatic activities can vary depending on the specific substrate.

All samples of egg whites were collected and diluted 10-fold by PBS. Initially, we studied the binding of the diluted egg white samples of various bird species to evaluate the working solutions of tracer **1** and tracer **2**. The concentration of the substrate in the assay tube was determined to be 3 nM based on fluorescence intensity measurements. The FP signal was recorded immediately after mixing the tracers and egg white samples.

**Figure 2:**
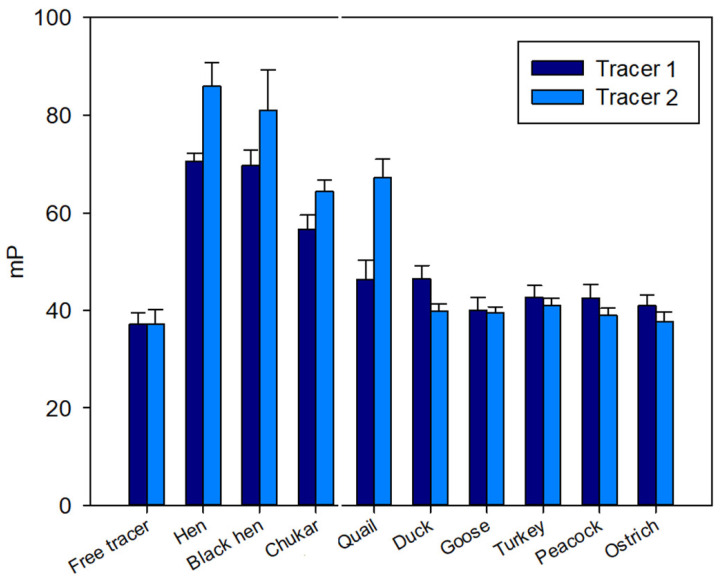
FP signal changes upon interaction of tri- (**1**) and pentasaccharide (**2**) tracers with lysozyme of different bird species. Final concentration of tracers **1** and **2** is 3 nM, 25 °C, pH 7.4.

A pronounced initial increase in the FP signal was observed for samples from hen, black hen, chukar, and quail eggs (Figure 2), exhibiting a response similar to that observed for the hen egg white [27]. In contrast, for the remaining samples (duck, turkey, peacock, goose, and ostrich) the FP signal remained at the level of free tracers **1** and **2** (Figure 2).

**Figure 3:**
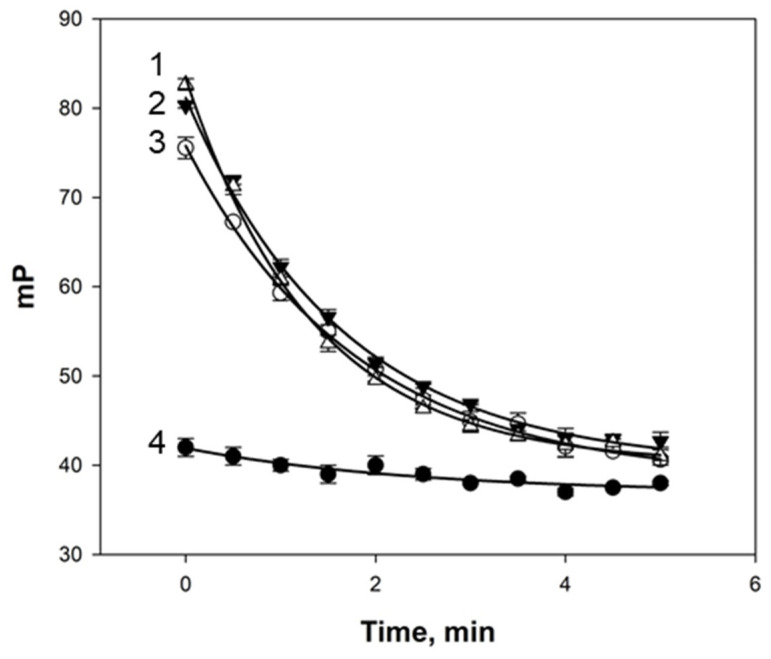
Kinetics of FP signal change after mixing samples of quail (1), chukar (2), hen (3), and duck (4) eggs with tracer **2** (3 nM) at 25 °C and pH 7.4.

The kinetics of the interaction between the egg white samples and tracers **1** and **2** were subsequently investigated. It was observed that, following binding to tracer **1**, the FP values remained unchanged for all egg white samples, whereas after binding to tracer **2**, the FP values progressively decreased over time (Figure 3).

**Figure 4:**
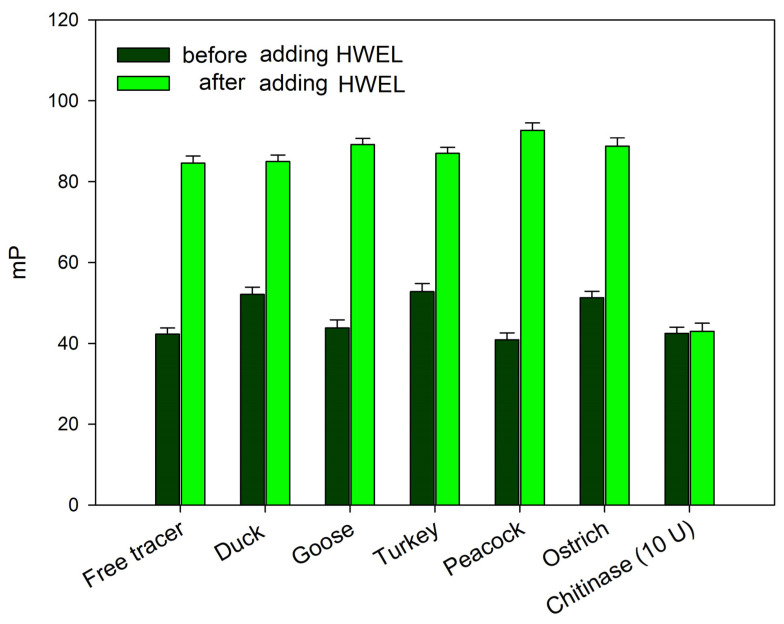
FP signal changes upon the interaction of tracer **2**, pre-incubated with egg white samples from different bird species, following the addition of the HEWL (final concentration of 100 µg/mL) to the reaction mixture. Final concentration of tracer **2** is 3 nM (25 °C, pH 7.4).

The absence of changes in the fluorescence polarization signal upon the addition of bird egg samples (duck, turkey, peacock, goose, and ostrich) may indicate either a complete lack of interaction with synthetic chitooligosaccharides **1** and **2** or their degradation—a phenomenon previously observed under the action by chitinases. In order to exclude the possibility of the rapid cleavage of the chitooligosaccharide chain without the formation of a stable complex (as observed with chitinases), the following experiment was conducted. The samples of egg whites from the duck, goose, turkey, peacock, and ostrich were added to working solutions of tracer **2**. After a 10 min incubation, the hen egg white lysozyme (HEWL) was added to the test tube (for a final concentration of 100 μg/mL). A sharp increase in the FP signal followed by a decrease over time was observed, similarly to the response for a pure HEWL solution (Figure 4). These results indicate that tracer **1** remains unaffected by egg proteins from these birds. Additionally, the analyzed samples do not contain inhibitors that interfere with the interaction between lysozyme and the chito-oligosaccharide chain. Therefore, the observed difference in the FPA between hen, black hen, chukar, and quail eggs on one hand and duck, goose, turkey, peacock, and ostrich eggs on the other, is likely attributable to the distinct nature of lysozymes, which differ fundamentally in their substrate specificity.

**Figure 5:**
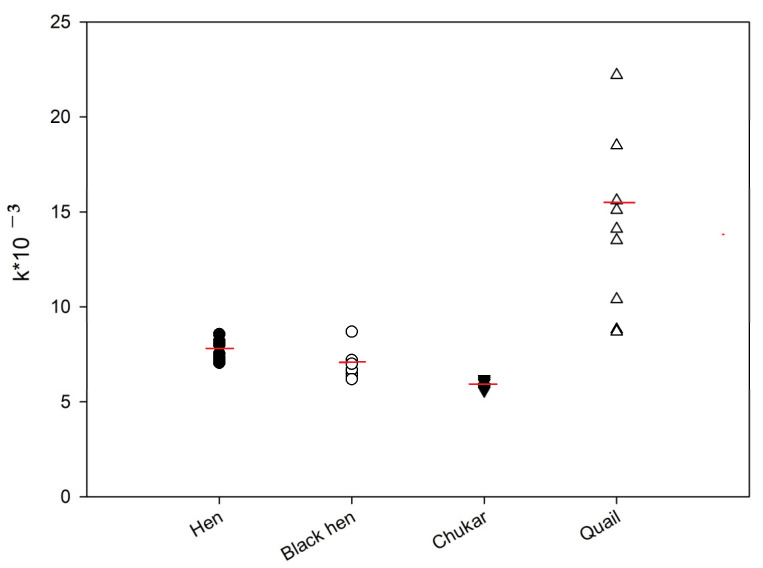
Lysozyme activity in egg samples from different birds. Final concentration tracer **2** is 3 nM, dilution of egg white samples is 1:100. Red line shows median value.

The kinetics of the FP signal were subsequently analyzed for the protein samples from hen, black hen, partridge, and quail eggs. Figure 2 shows representative FP signal changes for these samples. As illustrated, after reaching a maximum FP signal, a gradual decrease was observed over time, suggesting a substrate degradation by the lysozyme present in the egg protein [19]. These kinetic data were approximated using Equation (1), and the rate constants (k) were calculated. The activity of lysozyme in egg samples was compared using the k values (Figure 5). The ANOVA analysis revealed that the differences between the hen and black hen were not statistically significant, whereas all other groups exhibited significant differences (*p* < 0.05). The chitinase activity of quail eggs is much higher if compared to other samples. However, significant variability was observed in the data for these samples.

A slight decrease in the chitinase activity of lysozyme was observed in the row hen—black hen—chukar egg samples. Also, low variability was detected among these samples.

## 4. Discussion

It is known that two radically different types of lysozymes are found in the egg whites of birds. Type C is present in high concentrations in the egg white of the domestic chicken and many other species of the Galliformes order, as well as in the egg whites of some species of the waterfowl order Anseriformes [28,29]. Type-G lysozyme has been detected and characterized in the egg white of the domestic goose (*Anser anser*) [30,31]. In the egg whites of certain waterfowl species, such as geese and swans, both the forms C and G of lysozyme occur simultaneously [32]. Both type-C and type-G lysozymes are muramidases, but they differ markedly in their amino acid sequence, molecular weight, and enzymatic properties [29,33,34,35]. Additionally, these two types of lysozyme are completely distinct antigenically: antibodies to type-G lysozyme do not cross-react with type-C lysozyme, and vice versa [36].

Chicken and goose lysozymes differ in their molecular weight, amino acid composition (in particular, in cysteine and tryptophan contents), primary structure, and enzymatic properties, such as the optimal pH, sensitivity to ionic strength, and, especially, sensitivity to inhibitors like N-acetylglucosamine (GlcNAc) (Table 1) [37].

The main biological role of lysozyme in eggs is to exhibit bacteriostatic or lytic activity. However, its content varies significantly across the eggs of many bird species (Table 2). In terms of the lysozyme content in protein, chicken eggs rank first, followed in descending order by the eggs of turkeys, guinea fowl, ducks, and geese [31,33,34,35,37,38]. The lysozyme content also varies among the different protein layers of the egg. There is a tendency for the lysozyme content to increase from the inner layer to the outer layers.

Due to the presence of lysozyme as a natural antibiotic, the egg whites exhibit bactericidal properties, which are critical for protecting the developing embryo from infection. Lysozyme constitutes approximately 4% of the total egg white proteins. It is known that eggs with high levels of lysozyme have denser proteins and a greater pH difference between the egg yolk and egg white. A positive correlation with egg hatchability has also been established [45,46]. The hatchability was 69.5% with the lysozyme level of 4.68–4.92 mg/mL, 82.1% with the level of 5.0–5.9 mg/mL, and 84.8% with the level of 6.0–6.42 mg/mL. 

An increase in the lysozyme concentration enhances the immunobiological properties of eggs. It is assumed that lysozyme levels in the egg white are genetically inherited. Significant variations in lysozyme content have been observed in eggs laid by hens of the same species [47]. The direct selection for lysozyme content may improve protein quality and lead to an increase in lysozyme levels in egg whites. Eggs with a high lysozyme activity in the protein retain their quality longer during storage [48]. Therefore, assessing lysozyme activity [49] is crucial for characterizing the egg quality, and it is essential to accurately measure its enzymatic activity.

One of the methods for assessing lysozyme activity is the turbidimetric assay of bacterial cell lysis [50]. *Micrococcus luteus* is used for this purpose because it is a Gram-positive bacterium, making it susceptible to lysozyme, and because these cells are easy to culture. Another approach uses live bacteria, such as *Micrococcus lysodeikticus* [16]. In this case, lysozyme catalyzes the hydrolysis of *M. lysodeikticus* by the diffusion into agar, resulting in the formation of a visible transparent ring. While this method is relatively simple, it is not very accurate and requires a long time, often more than 10 h. Enzyme activity can also be measured turbidimetrically, nephelometrically, or fluorometrically in the liquid phase. However, only a few methods are currently known for determining enzyme activity by fluorometry [51,52].

Our proposed method for measuring the lysozyme enzymatic activity by the FPA with fluorescently labeled oligosaccharide derivatives [26,27] offers a promising approach for characterizing eggs. This method allows for the determination of the active lysozyme content in egg whites, which is not only crucial for predicting the duration of egg storage but also for estimating the hatchability of chicks in an incubator. Given the heritability of lysozyme content, this selection method could be useful for identifying promising birds for breeding.

**Figure 6:**
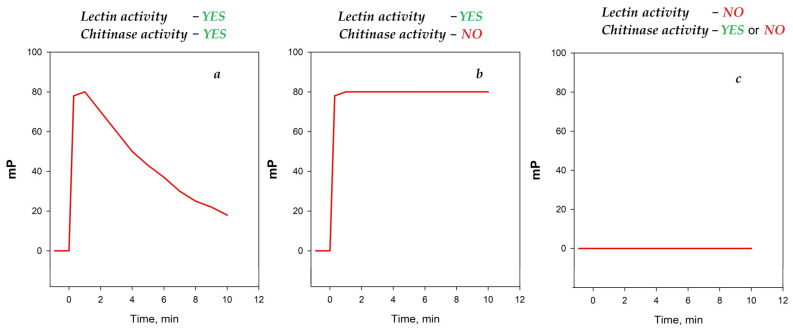
The principal types of time-dependent FP signal behaviors observed upon mixing chito-oligosaccharide tracer solutions with egg white samples. (**a**) the result of the addition of the egg white samples to tracer suggests the formation of a stable complex (manifestation of lectin activity), followed by the subsequent degradation of chito-oligosaccharide tracers (manifestation of chitinase activity); (**b**) the result of the addition of the egg white samples to the tracer indicates a complex formation without a subsequent enzymatic degradation (only lectin activity); (**c**) the results of addition of the egg white samples to tracer indicates the absence of lectin activitie.

Upon mixing the solutions of chito-oligosaccharide tracers with egg white samples from various bird species, different types of time dependencies of the FP signal were observed (Figure 6). Thus, upon the addition of the egg white samples from hens, black hens, chukars, and quails to tracer **2**, a rapid initial increase followed by a gradual decline in the FP signal was observed (Figure 6a). This type of behavior suggests the formation of a stable complex (manifestation of lectin activity), followed by the subsequent degradation of chito-oligosaccharide tracers (manifestation of chitinase activity). Upon the addition of the hens, black hens, and chukars egg white samples to the shorter trisaccharide tracer **1**, a rapid increase in the FP signal was observed, followed by a stable plateau (Figure 6b). This type of behavior indicates a complex formation without a subsequent enzymatic degradation.

The egg white sample from quails exhibited the first type of dependency (Figure 6a) when interacting with tracer **2** and no significant changes in the FP signal when interacting with tracer **1**. This similar behavior has been shown previously for human lysozyme [26]. Notably, egg white samples from ducks, geese, turkeys, peacocks, and ostriches exhibited no significant changes in the FP signal upon interaction with either tracer **1** or tracer **2** (Figure 6c). This type of behavior can be related to the absence of any interaction between chito-oligosaccharides and is also observed for “classical” chitinases that rapidly cleave the substrate without forming a stable complex. The incubation of these samples with tracer **2** followed by the addition of the HEWL, which is known to bind to the chitooligosaccharide sequence, did not reveal the cleavage of the tracer (see Figure 4). This confirms the absence of an interaction between chito-oligosaccharide tracers and lysozymes from the egg whites of ducks, geese, turkeys, peacocks, and ostriches.

Overall, our findings suggest that the interaction between chito-oligosaccharides and lysozymes varies fundamentally among different avian species. This distinction enables the further profiling and characterization of the lysozyme activity in egg white samples.

A comparative analysis of the lysozyme amino acid sequences from chicken, turkey, peacock, duck, and quail egg whites—all representing the C-type lysozyme class—reveals intriguing variations in enzymatic activity [36]. While these lysozymes maintain a conserved tertiary structure, crucial for their function as bacterial cell wall hydrolases, subtle differences in their primary amino acid sequences significantly impact their catalytic efficiency. This study focused on understanding these differences to explain observed variations in activity levels between species. Remarkably, the catalytic residues, Glu-35 and Asp-52, and their immediate neighboring amino acids remain invariant across all five species [53]. This conservation underscores their vital role in the lysozyme mechanism, which involves the binding and hydrolysis of β-(1→4)-glycosidic bonds in peptidoglycan [12,13,14,15]. The key differences lie in the amino acid substitutions at positions 15, 41, and 121, as highlighted in Table 3. These positions are located in regions potentially influencing substrate binding or conformational flexibility, rather than directly in the catalytic site itself.

Specifically, the substitution at position 15 stands out. Chicken lysozyme features a histidine residue at this position, a positively charged amino acid [56]. In contrast, turkey, peacock, and duck lysozymes exhibit a leucine residue—non-polar and hydrophobic. This substitution is strongly correlated with observed differences in the activity against a fluorescently labeled oligosaccharide substrate. The altered hydrophobicity at position 15 likely affects the substrate binding affinity or the enzyme’s overall conformation, impacting its catalytic efficiency [56]. Further investigation using techniques like X-ray crystallography or molecular dynamics simulations could provide a more detailed understanding of these conformational changes [57]. Positions 41 and 121 also show variability. Peacock and turkey lysozymes exhibit a histidine residue at both positions, introducing additional positive charges compared to the chicken lysozyme. Conversely, duck lysozyme shows substitutions with glutamine (Gln) and serine (Ser), polar but uncharged residues. These variations suggest a complex interplay of electrostatic interactions and hydrophobicity that influences enzyme–substrate interactions and, ultimately, the overall catalytic rate. Future research might explore the impact of ionic strength and pH on the activity of these different lysozymes, given the influence of charged amino acid residues. Furthermore, a broader phylogenetic analysis incorporating other avian species could reveal evolutionary patterns and selection pressures shaping lysozyme sequence variation. Such a comprehensive analysis could help clarify the functional implications of these amino acid substitutions and potentially reveal a correlation between lysozyme activity and the specific bacterial challenges faced by each bird species.

The literature’s data have shown that lysozyme in quail eggs exists in two protein types, A and B (QEWL A and QEWL B). When separated by ion exchange chromatography, these enzymes exhibited the same optimal pH profile for lytic activity, with a broad pH range (pH 5.0–8.0), but differed in their mobility on native PAGE. The amino acid sequence of lysozyme A from quail egg protein (QEWL A) was identical to the quail lysozyme described by Kaneda et al. [34], with six amino acid substitutions compared to chicken egg lysozyme: at positions 3 (Phe to Tyr), 19 (Asn to Lys), 21 (Arg to Gln), 102 (Gly to Val), 103 (Asn to His), and 121 (Gln to Asn). One substitution common to both QEWL A and QEWL B occurred at position 21, where Gln was replaced by Lys. Additionally, QEWL B displayed a leucine insertion between positions 20 and 21. This was the first report identifying QEWL B as having 130 amino acids. Despite these amino acid differences, the antigenic determinants were similar between QEWL A and QEWL B, as both enzymes were recognized by polyclonal antibodies raised against the HEWL. However, these variations could potentially influence the enzymatic activity of QEWL A and QEWL B. The variation in lysozyme activity values observed for quail eggs in this study (Figure 5) may be attributed to differences in the relative proportions of the two lysozyme types among individual birds.

## 5. Conclusions

Lysozyme, an enzyme with potent antibacterial properties, exhibits considerable interspecies variation in its ability to interact with short chitin-related oligosaccharides. Our research demonstrates a significant difference in the lysozyme activity, specifically its ability to cleave chitooligosaccharides, amongst various avian species. This variation is effectively profiled using a fluorescence polarization (FP) assay employing fluorescently labeled chitopentaoside which consisted of five GlcNAc units. This method offers a sensitive and high-throughput approach to lysozyme quantification, even within complex mixtures like food products. We found that the FP assay employing chitooligosaccharide tracers effectively measures the lysozyme activity in the eggs of several bird species. Specifically, hen, black hen, chukar partridge, and quail eggs demonstrated significant lysozyme activity against the fluorescently labeled chitopentaoside. Quail eggs exhibited the highest enzymatic activity, surpassing that of both commercially available chicken eggs and black hen eggs, which showed comparable levels. Chukar partridge eggs displayed a slightly lower activity. This variation likely stems from subtle amino acid sequence differences in the lysozyme protein itself, impacting substrate binding affinity and catalytic efficiency. Furthermore, post-translational modifications, such as glycosylation, can also affect the enzyme’s activity. These modifications vary between species and even within individuals, contributing to the observed variability.

Conversely, egg whites from ducks, geese, turkeys, peacocks, and ostriches showed no detectable interaction with the chitooligosaccharide tracer, suggesting a significant structural divergence in their lysozyme isoforms. This lack of activity could be attributed to several factors, including substantial sequence variations in the lysozyme active site or the presence of inhibitory compounds within the egg white matrix. Further investigation employing techniques like mass spectrometry and protein sequencing could elucidate the precise structural differences underpinning these functional discrepancies. The ability to accurately quantify lysozyme activity using this FP-based method has significant implications. For instance, selecting eggs with a high lysozyme content allows for improved egg preservation, as lysozyme contributes to inhibiting bacterial growth and extending shelf life. Moreover, eggs with elevated lysozyme levels may correlate with improved hatchability rates in avian species, potentially reflecting a stronger innate immune system in the developing embryo. Therefore, this assay provides a valuable tool for both food science applications and avian reproductive biology research, enabling the selection of eggs with superior qualities for both commercial and scientific purposes. The implications extend beyond simple egg selection; understanding the diversity of the lysozyme function across species could even inform the development of novel antimicrobial agents inspired by avian lysozymes with an enhanced activity against specific pathogens.

## Figures and Tables

**Table 1:** Some important differences between lysozymes of types C and G [37].

	Lysozymes	
	C Type (Hen)	G Type (Goose)
Molecular Weight, Da	14,500	20,500
Cys Content (residues/mole)	8	4
Trp Content (residues/mole)	6	3
Specific Activity	1	6 ± 0.5
Inhibition by GlcNAc	high	almost none

**Table 2:** Some sources of bird lysozyme.

Source of Lysozyme	Type	Amino Acid	Amount of Lysozymein Egg Whites	References
Chicken egg white	c	129	2500–3500 µg/mL	[38]
Duck egg white	c	130	1000–1300 µg/mL	[38]
Pheasant egg white	c	129	ND *	[39,40]
Partridge egg white	c	129	ND *	[41]
Turkey egg white	c	129	ND *	[42]
Peacock egg white	c	129	ND *	[32]
Quail egg white	c	129,130	ND *	[43,44]
Goose egg white	g	181	500–700 µg/mL	[38]
Ostrich egg white	g	178	180 mg–530 mg/kg	[38]

* ND—not detected.

**Table 3:** Differences between amino acid residues in C-type lysozymes.

Lysozyme Has Activity to Tracer 2	Lysozyme Has No Activity to Tracer 2		
Hen and quail [54]	Turkey [54]	Peacock [54]	Duck [55]
His-15	Leu-15	Leu-15	Leu-15
Gln-41	His-41	His-41	Gln-41
Gln-121	His-121	His-121	Ser-121

## Data Availability

The original contributions presented in this study are included in the article. Further inquiries can be directed to the corresponding authors.
